# BET-Inhibitors Disrupt Rad21-Dependent Conformational Control of KSHV Latency

**DOI:** 10.1371/journal.ppat.1006100

**Published:** 2017-01-20

**Authors:** Horng-Shen Chen, Alessandra De Leo, Zhuo Wang, Andrew Kerekovic, Robert Hills, Paul M. Lieberman

**Affiliations:** The Wistar Institute, Philadelphia, PA, United States of America; University of North Carolina at Chapel Hill, UNITED STATES

## Abstract

Kaposi’s Sarcoma-associated Herpesvirus (KSHV) establishes stable latent infection in B-lymphocytes and pleural effusion lymphomas (PELs). During latency, the viral genome persists as an epigenetically constrained episome with restricted gene expression programs. To identify epigenetic regulators of KSHV latency, we screened a focused small molecule library containing known inhibitors of epigenetic factors. We identified JQ1, a Bromodomain and Extended Terminal (BET) protein inhibitor, as a potent activator of KSHV lytic reactivation from B-cells carrying episomal KSHV. We validated that JQ1 and other BET inhibitors efficiently stimulated reactivation of KSHV from latently infected PEL cells. We found that BET proteins BRD2 and BRD4 localize to several regions of the viral genome, including the LANA binding sites within the terminal repeats (TR), as well as at CTCF-cohesin sites in the latent and lytic control regions. JQ1 did not disrupt the interaction of BRD4 or BRD2 with LANA, but did reduce the binding of LANA with KSHV TR. We have previously demonstrated a cohesin-dependent DNA-loop interaction between the latent and lytic control regions that restrict expression of ORF50/RTA and ORF45 immediate early gene transcripts. JQ1 reduced binding of cohesin subunit Rad21 with the CTCF binding sites in the latency and lytic control regions. JQ1 also reduced DNA-loop interaction between latent and lytic control regions. These findings implicate BET proteins BRD2 and BRD4 in the maintenance of KSHV chromatin architecture during latency and reveal BET inhibitors as potent activators of KSHV reactivation from latency.

## Author Summary

KSHV is an oncogenic human herpesvirus implicated as the causative agent of KS and cofactor in pleural effusion lymphomas (PELs). The latent virus persists in PELs as an epigenetically regulated episome. We found that small molecule inhibitors of BET family have potent activity in triggering the lytic switch during latent infection in PELs. The BET family inhibitor JQ1 disrupted the latent virus from maintaining a closed DNA loop conformation. These findings have implications for treatment of KSHV-associated malignancies with epigenetic modulators of the BET inhibitor family.

## Introduction

Kaposi’s Sarcoma-associated Herpesvirus (KSHV) is a human gammaherpesvirus responsible for all forms of Kaposi’s Sarcoma (KS) and strongly associated with pleural effusion lymphomas (PELs) and Castleman’s Disease[1]. KSHV can establish long-term latent infection in B-lymphocytes where it persists as a stable, chromatin-associated circular minichromosome, commonly referred to as an episome [2, 3]. During latent infection, the viral genome expresses only a few viral genes required for maintaining the latent state and host-cell survival [4, 5]. The major latency transcripts include the multi-cistronic RNAs encoding LANA (ORF73), vCyclin (ORF72), vFLIP (ORF71), K1, and 21 miRNAs. The major immediate early genes are also regulated as a cluster of RNAs that can be initiated during the early stage of the reactivation process. These include the immediate early transcriptional activator RTA (ORF50), KbZip (ORF51), and a series of transcripts that are made in the opposite orientation that include ORF45-49. Lytic transcription is repressed during latency, while latency transcription occurs efficiently. How these regions are differentially regulated and how they communicate with each other remains an area of active interest.

KSHV latency is maintained by several epigenetic regulatory mechanisms. Lytic cycle regulatory regions, especially the immediate early promoter regions controlling RTA transcription are regulated by bivalent histone modifications that include both euchromatic H3K4me3 and repressive H3K27me3 at the same regulatory locus [6, 7]. Inhibitors of polycomb-associated H3K27me3 methyltransferase EZH2 are sufficient to induce lytic cycle replication [8–10]. In KSHV positive B-cell pleural effusion lymphomas, KSHV latency can be reactivated by other epigenetic pathways, including histone deacetylase (HDAC) inhibitors in combination with phorbol esters [11]. Lytic reactivation may also be induced by other cellular stress pathways, including hypoxia [12], reactive oxygen species (ROS)[13], cytokine stimulation[14], and terminal differentiation[15].

During latent infection in PEL cells, the KSHV genome is also regulated by higher-order epigenetic regulatory mechanisms [16]. We have shown that the chromatin organizing factor CTCF colocalizes with cohesins at several locations in the KSHV genome, including the latency control region [17]. Subsequent studies revealed that KSHV latency control region formed a DNA-loop interaction with the lytic control region, mediated in part by the CTCF-cohesin complex [18]. Chromosome conformation capture (3C) revealed that the control regions for the lytic and latent cycle transcripts are in close proximity during latency, and that this is disrupted during the reactivation process. Depletion of cohesin subunits, including RAD21, SMC1, or SMC3 led to the reactivation of KSHV [19]. Depletion of CTCF, as well as Rad21, were also found to be restriction factors for KSHV lytic reactivation, especially when combined with HDAC inhibitor sodium butyrate [20]. CTCF is a sequence-specific DNA binding protein that has multiple functions in gene regulation, including the formation of chromatin boundaries and DNA-loop interactions [21]. Cohesins form a ring-like structure that mediates DNA-DNA interactions important for sister chromatid cohesion, homologous recombinational DNA repair, and promoter-enhancer communication for transcriptional regulation [22]. How other factors may regulate the formation and dissolution of these higher-order DNA structures, and how these impact gene expression patterns is not completely known.

The KSHV latent episome is maintained largely by the viral-encoded protein LANA [23, 24]. LANA is a sequence-specific DNA binding protein that interacts specifically with GC-rich elements in the terminal repeats (TR) of KSHV [25]. LANA binding to the TR is necessary for tethering the viral episome to metaphase chromosomes, and can also function as an efficient origin of DNA replication [24, 26, 27]. LANA can also regulate transcription of viral and host genes, and interact with other regions of the viral and cellular chromosome, but with lower affinity than at the viral TR [28, 29]. LANA interacts with many host proteins. Among these are the Bromodomain 2 (BRD2) and BRD4 members of the BET family [30–33]. Bromodomains are known to interact with acetylated lysines, most typically found on histone tails in euchromatic regions [34, 35]. BRD2 and 4 have been implicated in the regulation of several viruses, including tethering and transcriptional regulation of HPV through the E2 protein, a viral orthologue of KSHV LANA [36–38]. The precise function of BRD2 or BRD4 in mediating LANA function is not clear, but several lines of evidence suggest that these factors facilitate LANA-dependent episome maintenance and transcription regulation.

Small molecule inhibitors of BET proteins, especially BRD2 and BRD4, have been highly effective at disrupting their biochemical function in binding acetylated lysines on histone tails [39, 40]. A prototype BET inhibitor, JQ1, has been shown to effectively inhibit transcription mediated by BRD2 and BRD4 dependent super-enhancers, including those regulating the cMyc oncogene in several cancer models [40, 41]. JQ1 has also been shown to trigger the reactivation of latent HIV [42–46]. The mechanism of HIV reactivation by JQ1 is thought to be through the redistribution of BRD2 and BRD4 to promote RNA polymerase elongation on HIV genomes [43, 45]. The effects of BET inhibitors on KSHV latency is not as well understood. One study found that BET inhibitors synergize with lenalidomide to selectively kill KSHV positive PEL cells [47]. Here, we screened a library of small molecule epigenetic modulators and found BET inhibitors to be among the most potent for activation of KSHV lytic reactivation, and further investigate the mechanism of their action.

## Results

### Identification of BET-inhibitors as disruptors of KSHV latency

**Fig 1:**
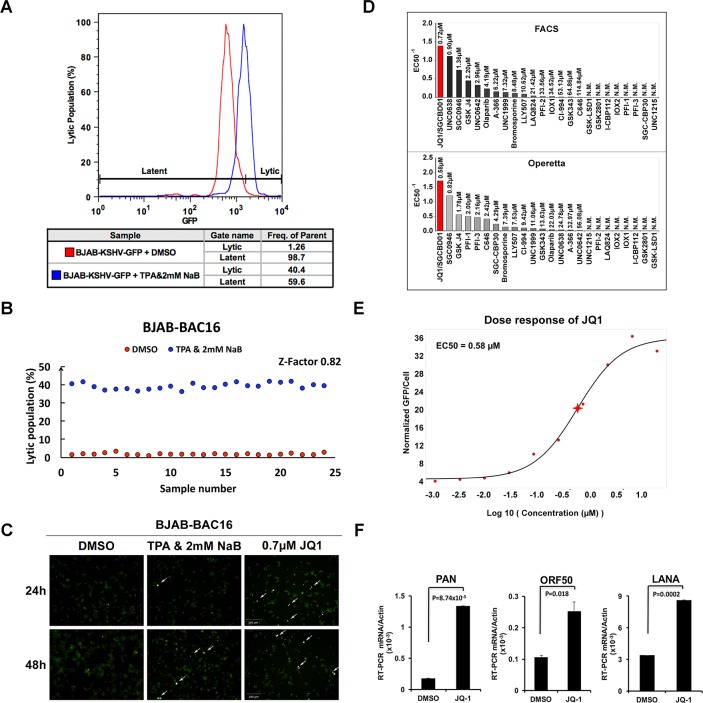
Identification of JQ1 in a high-throughput screen for epigenetic regulators of KSHV lytic reactivation. **(A)** BJAB-BAC16 cells were assayed by FACS for GFP fluorescence intensity after 48 hr incubation with 1% DMSO (red) or TPA+2mM NaB (blue). **(B**) Z-factor calculation for high-throughput FACS analysis of KSHV lytic population. **(C)** Operetta high-content image analysis for GFP intensity in BJAB-BAC16 cells treated for 24 or 48 hr with 1%DMSO (left panels), TPA+2 mM NaB (middle panel), or 0.7 μM JQ1(right panel). **(D)** Summary of high-throughput screening using FACS (top panel) or Operetta (lower panel). Compound activity was ranked from left to right based on the reciprocal of the EC50 value calculated for each compound. **(E)** EC50 calculation for JQ1 using Operetta high-content imaging. **(F)** RT-qPCR for PAN, ORF50, and LANA transcripts relative to cellular actin in BJAB-BAC16 cells treated for 24 hrs with 1% DMSO or 4 μM JQ1. Error bars represent standard deviation from mean (sdm) and p-values were calculated by 2-tailed student t-test.

To identify epigenetic regulators of KSHV latent to lytic switch, we developed a simple reporter cell line, BJAB-BAC16, consisting of BJAB cells stably infected with KSHV Bac16 that carry a constitutively expressed GFP gene. BJAB-BAC16 cells were first tested for their ability to respond to known lytic cycle reactivating reagents consisting of phorbol ester TPA combined with histone deacetylase inhibitor sodium butyrate (NaB) (Fig 1). Addition of TPA with NaB led to a robust stimulation of GFP signal from BJAB-BAC16 cells using FACS (Fig 1A), as well as by high-content imaging using an Operetta (Fig 1C). This indicated that GFP signal could be used as a surrogate for lytic cycle gene activation. Both assays were miniaturized for 384-well high-throughput screening with 25,000 cells per well and demonstrated robust statistical properties based on Z-factor >0.8 (Fig 1B). We then proceeded to screen a focus library of 24 compounds with known epigenetic targets. Each compound was arrayed for a 10-point dose response curve and an EC50 value was calculated for stimulation of GFP signal (Fig 1D). We found that several compounds stimulated KSHV lytic cycle with EC50 < 3 μM, while only one compound, the BET inhibitor JQ1, activated GFP signal at EC50 <1 μM in both FACS and Operetta assays. Using the high-content analysis of the Operetta, JQ1 was found to have an EC50 of 0.58 μM (Fig 1E). The effect of JQ1 on KSHV lytic cycle gene expression was validated by RT-qPCR for KSHV PAN, ORF50, and LANA (Fig 1F).

### JQ1 induces KSHV lytic gene transcripts and DNA replication in latently infected PEL cells

**Fig 2:**
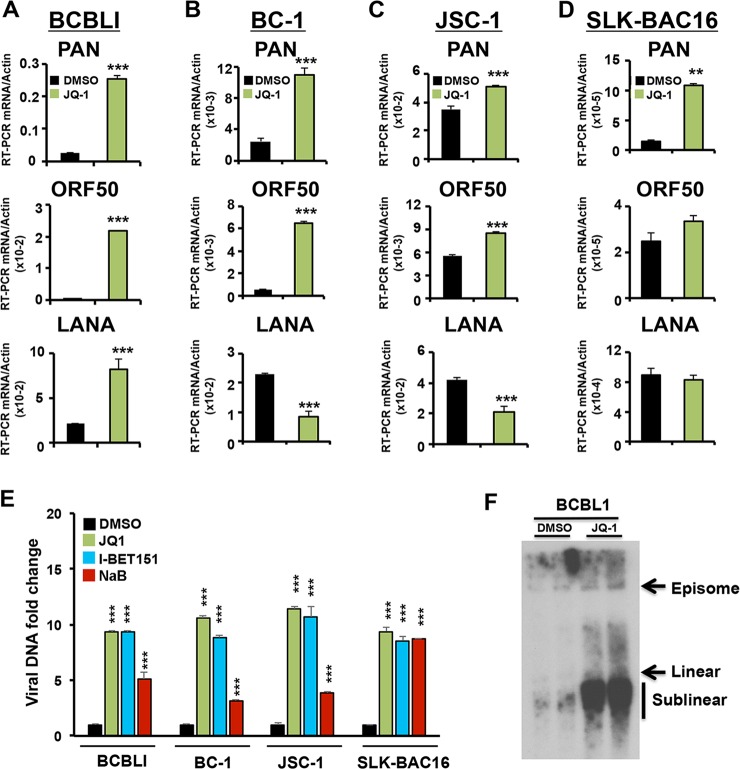
JQ1 induces KSHV lytic transcription and DNA replication in latently infected PEL cells. **(A)** RT-qPCR for PAN, ORF50 or LANA in BCLB1 cells treated daily for 72 hrs with 1% DMSO or 4 μM JQ1. **(B)** RT-qPCR as in panel A for BC-1 cells **(C)** JSC-1 cells or **(D)** SLK-BAC16 cells. ** P < 0.01 *** P < 0.001. **(E)** qPCR of KSHV genome in different cell lines treated as in (A) with DMSO, 4 μM JQ1, or 4 μM I-BET151, or 2mM NaB. The data are expressed as fold change of the treated versus untreated (DMSO) cells. *** P < 0.001 **(F)** PFGE analysis of BCBL1 cells treated as in panel A, and detected by Southern blot with probe specific for KSHV DNA. Electrophoretic positions for viral episomes, linear, and sublinear genomes are indicated.

We next tested JQ1 for its ability to activate KSHV lytic cycle in various KSHV-positive cell lines. BCBL1, BC-1, JSC-1, or SLK-BAC16 were treated with 4 μM JQ1 daily for 3 days (72 hrs) and then assayed by RT-qPCR for transcription of PAN, ORF50, and LANA (Fig 2A–2D). We found that JQ1 efficiently activated viral lytic genes for PAN and ORF50 in all PEL cell lines (Fig 2A–2C). JQ1 efficiently stimulated PAN in the non-lymphoid cell line SLK-BAC16, but had less significant activation on ORF50 (Fig 2D). The effects of JQ1 on LANA transcripts were also cell-dependent, with an ~ 4 fold increase in BCLB1, a ~2 fold decrease in BC-1, and no change in JSC-1 or SLK-BAC16 (Fig 2A–2D, lower panels). We next tested whether JQ1, and another BET inhibitor I-BET151, could induce KSHV DNA replication (Fig 2E). Viral DNA replication was monitored by qPCR by comparing viral relative to cellular DNA. We found that JQ1, as well as I-BET151, efficiently induced DNA replication in BCBL1, BC-1, JSC-1, and SLK-BAC16 cells. JQ1 and I-BET151 induced ~10 fold increase in viral DNA copy number, while positive control sodium butyrate (NaB) induced only 3–5 fold in PEL cells (Fig 2E). To further analyze the status of KSHV genomes after treatment with JQ1, we analyzed BCBL1 cells treated with DMSO or JQ1 by pulse-field gel electrophoresis (PFGE) followed by Southern blot analysis. We observed a large amplification of viral linear and sub-linear genomes (indicative of incomplete genome replication), confirming that lytic cycle DNA replication was activated by JQ1 (Fig 2F).

**Fig 3:**
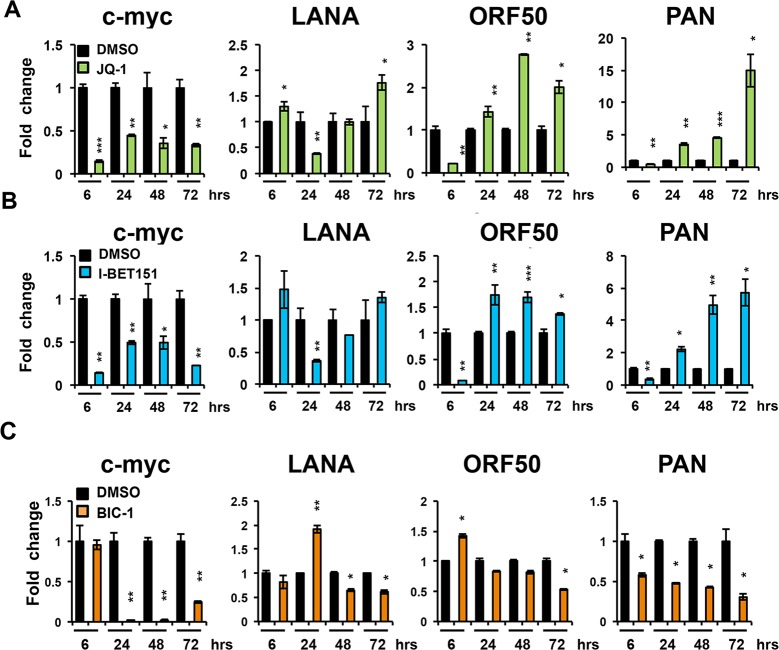
Time course of KSHV transcript response to various BET inhibitors. RT-qPCR for c-myc, LANA, ORF50 and PAN relative to actin in BCBLI cells treated with 1%DMSO, or 4uM JQ1 (A), 4uM IBET-151 (B) or 4uM BIC1 (C) for the indicated times. DMSO was used as control. The data are expressed as fold change of the treated versus untreated (DMSO) cells. * P < 0.05 ** P < 0.01 *** P < 0.001.

JQ1 is known to act rapidly on BRD2 and BRD4 binding to acetylated histones [41], but its kinetic effects on transcription can be complex. We determined the time course of KSHV transcripts after JQ1 treatment. As expected, cMyc transcription was reduced within 6 hrs after JQ1 treatment (Fig 3A). Interestingly, KSHV transcripts for lytic cycle (ORF50 and PAN) were also reduced at 6 hrs, while LANA transcription was modestly elevated. However, by 72 hrs, KSHV lytic transcripts for ORF50 and PAN were 2 and 15 fold relative to DMSO control. Similar results were observed with another pan-BET inhibitor I-BET151 (Fig 3B). On the other hand, BIC-1, which is more selective for BRD2, had inhibited cMyc transcription by 24 hrs, but did not activate KSHV lytic gene transcription (Fig 3C). Similar observations were made with another PEL cell line BC3 (S1 Fig). These results indicate that structurally different pan-BET inhibitors can stimulate KSHV lytic transcription, but also raise the possibility that BET inhibitors may contribute indirectly to KSHV reactivation through suppression of myc or other cellular targets.

### Depletion of BRD4 or BRD2 reactivates lytic KSHV from latency

**Fig 4:**
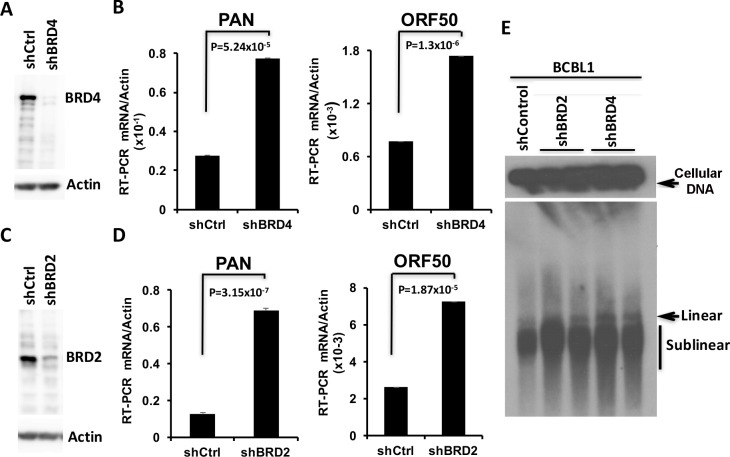
Depletion of BRD2 or BRD4 activates KSHV lytic cycle. **(A)** Western blot of BCBL1 cells transduced with shControl (shCtrl) or shBRD4, probed for BRD4 (top) or Actin (bottom). **(B)** RT-qPCR for PAN and ORF50 relative to actin in BCBL1 cells transduced with shCtrl or shBRD4. **(C)** Western blot of BCBL1 cells transduced with shCtrl or shBRD2 probed for BRD2 (top) or Actin (bottom). **(D)** RT-qPCR for PAN and ORF50 relative to actin in BCBL1 cells transduced with shCtrl or shBRD2. **(E)** PFGE analysis of BCBL1 cells transduced with shCtrl, shBRD2 (in duplicate), or shBDR4 (in duplicate) as indicated, and assayed by Southern blot with KSHV genome probe.

To determine whether BRD2 or BRD4 were the targets of JQ1-mediated reactivation of KSHV, we transduced BCBL1 cells with lentivirus expressing shBRD2, shBRD4, or shControl (shCtrl) (Fig 4). shBRD4 reduced BRD4 efficiently (Fig 4A), and produced a ~2.5 fold increase in PAN and 2 fold increase in ORF50 (Fig 4B). Similarly, shBRD2 partially reduced BRD2 expression (Fig 4C), and led to a ~6 fold increase in PAN and ~2.5 fold increase in ORF50 (Fig 4D). Both shBRD4 and shBRD2 produced a modest (~2 fold) increase in linear and sublinear KSHV genomes in PFGE analyses (Fig 4E). These results indicate that depletion of either BRD4 or BRD2 can partially induce KSHV lytic cycle transcription, and weakly induce lytic DNA replication.

### BRD2 and BRD4 binds regulatory regions of KSHV latent episome

**Fig 5:**
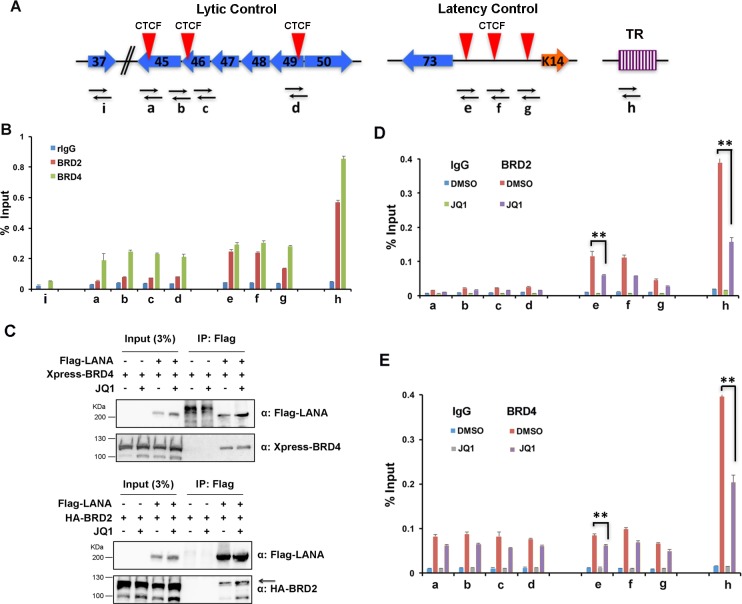
Interaction of BRD2 and BRD4 with KSHV latent episomes. **(A)** Schematic of KSHV genome regulatory regions tested by ChIP assay and primer positions a-i. Red triangles represent CTCF binding sites and TR is terminal repeats. Open reading frames for KSHV gene are indicated. The lytic control region (primers a-d) is considered the region between ORF45 and ORF50. The latency control region (primers e-g) is considered as the region encompassing ORF75 to K14. The terminal repeat (TR) contains high affinity LANA binding sites (primer h). **(B)** ChIP assay in BCBL1 cells with antibodies to BRD2 (red), BRD4 (green), or control IgG (blue) at primer position a-i as indicated. **(C)** CoIP assay for FLAG-LANA interaction with Xpress-BRD4 (top panel) or HA-BRD2 (lower panel) in the presence or absence of 4 μM JQ1. 293 cells were transfected with FLAG-LANA or FLAG-Vector and treated with 4 μM JQ1 or 1% DMSO for 24 hrs as indicated with input extract (left lanes) or after FLAG-IP (right lanes), as indicated. FLAG-IPs were then probed by Western blot with antibody to FLAG (LANA) or to Xpress (BRD4, top panel), or HA (BRD2, lower panel). **(D)** BCBL1 cells treated daily with 1% DMSO, or 4 μM JQ1 for 72 hrs were assayed by ChIP with antibody to BRD2 and assayed with primers a-h. **(E)** Same as in panel D, except with antibody to BRD4. Error bars are sdm and ** P < .01.

BRD2 and BRD4 are known to interact directly with LANA protein, but it is not known how they interact with the viral genome. We used Chromatin-Immunoprecipitation (ChIP) assay to measure the relative association of BRD2 and BRD4 with the KSHV genome at the LANA-binding site in the TR, and CTCF binding sites in latency control region and the lytic control region (Fig 5A). We found that BRD2 and BRD4 associated with all three viral genome positions, showing the highest enrichment at the LANA binding sites at the TR. BRD2 and BRD4 were found enriched similarly at the CTCF binding sites within latency control region, while BRD4 was selectively enriched at the lytic control region (Fig 5B). Neither BRD2 nor BRD4 bound to a region within the ORF37 gene (primer i), indicating that the enrichment at control regulatory regions is selective.

To determine if JQ1 affected BRD4 or BRD2 interaction with LANA, we assayed the effects of JQ1 on the coimmunoprecipitation (coIP) of LANA with BRD4 (Fig 5C, top panels) or with BRD2 (Fig 5C, lower panels). We found that addition of JQ1 did not disrupt, and had a modest stimulatory effect, on the interaction of LANA with BRD4 and BRD2 (Fig 5C). We next asked whether JQ1 had any effect on the binding of BRD4 or BRD2 proteins to KSHV genome by ChIP assay (Fig 5D and 5E). We found that JQ1 treatment reduced BRD2 and BRD4 interactions at the TR regions by ~50% (Fig 5D and 5E). BRD2 interactions at the latency control region were also reduced by ~50% by JQ1 (Fig 5D), while BRD4 interactions at the latency and lytic control regions were reduced by only ~20% after JQ1 treatment (Fig 5E). These findings indicate that BRD2 and BRD4 can interact with viral regulatory regions, and that JQ1 can partially disrupt these interactions.

### JQ1 causes a loss of RAD21, RNA pol II, and LANA from latency control region

**Fig 6:**
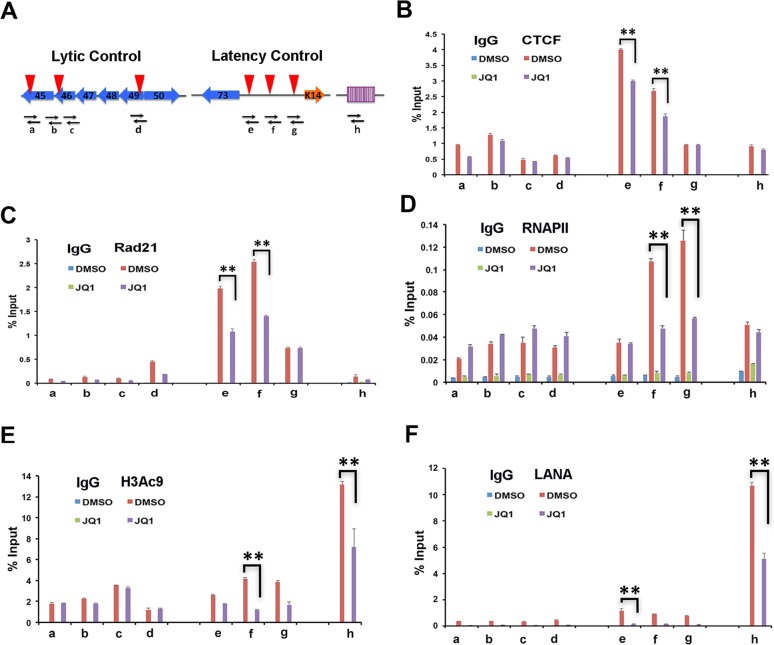
JQ1 alters epigenomic programming of KSHV latency control region. **(A)** Schematic of KSHV genome and primer positions a-h used for ChIP assays. **(B-F)** BCBL1 cells treated daily for 72 hrs with 1% DMSO or 4 μM JQ1 were assayed by ChIP for IgG or **(B**) CTCF, **(C)** Rad21, **(D)** RNAPII, **(E)** H3AcK9, or **(F)** LANA. Error bars are sdm and ** P < .01.

To better understand the mechanism through which JQ1 activates KSHV latent to lytic switch in BCBL1 cells, we performed ChIP assays for several key factors known to regulate this process, including CTCF, RAD21, RNAPII, histone H3K9ac, and LANA (Fig 6). JQ1 had a small inhibitory effect (~20% reduction) on CTCF binding at the latency control region (primers e-g), but no detectable changes in the lytic control region (primers a-d) or the TR (primer h) (Fig 6B). On the other hand, JQ1 reduced cohesin subunit Rad21 by 50% at the latency control region (primers e and f), as well as at the lytic control region (primer d) (Fig 6C). RNA polymerase II (RNAPII) was also reduced to 50% binding after JQ1 treatment at the latency control region (primer f and g), but slightly increased at the lytic control region (primers a-d). Similarly, histone H3 acetylated on K9 (H3Ac9) was reduced by 60% at the latency control region (primers f and g), and to a lesser extent that the TR, but did not change at the lytic control regions (Fig 6E). We also observed that LANA binding was reduced by ~50% at the TR, and by >80% at the latency control region and lytic control region after JQ1 treatment (Fig 6F). These findings indicate that JQ1 treatment alters the interaction with the latent KSHV genome for several key regulatory factors, including Rad21, RNAPII, and LANA. These findings also suggest that JQ1 leads to the selective loss of histone acetylation at the TR and latency control region, but not at the transcriptionally active lytic control region.

### JQ1 causes a loss of DNA looping between latent and lytic control regions

**Fig 7:**
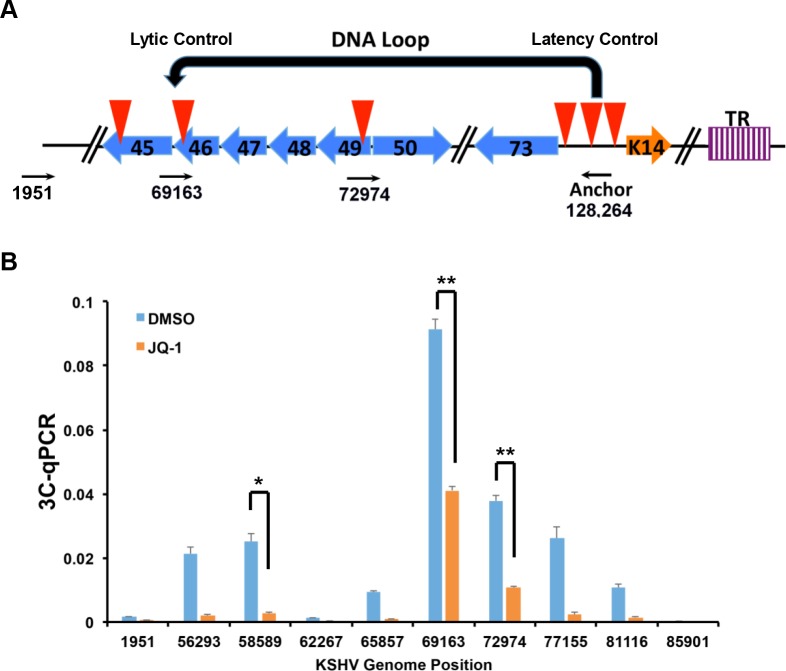
JQ1 inhibits DNA-loop formation between KSHV latent and lytic control regions. **(A)** Schematic of KSHV regulatory regions and primers used for 3C anchor at latency control region (128,264) and acceptor sites in lytic control region (69163), and others regions as indicated. **(B)** 3C assay in BCBL1 cells treated daily for 72 hrs with 1% DMSO or 4 μM JQ1 using anchor and acceptor primers, as indicated in **(A)**.

DNA-loop formation has been shown to occur between the latent and lytic control regions of KSHV in PEL cells [18], and disruption of this loop by depletion of Rad21 led to a reactivation from latency [19]. To determine whether JQ1 had any effect on KSHV DNA loop interactions, we performed chromatin conformation capture (3C) assays using an anchor primer at the KSHV latency control region (Fig 7A and 7B). As expected, we observed a selective interaction between the latent control region and the lytic control region (primer 69163) (Fig 7B). Treatment with JQ1 reduced this 3C interaction by 50%, as well as other weaker 3C interactions at positions downstream (56293 and 58589) and upstream (72974 and 77155) of the lytic control region. These findings suggest that JQ1 treatment alters the DNA conformation associated with stable episomal latency of KSHV.

To determine whether the effect of JQ1 chromatin factor binding was an indirect consequence of viral DNA replication, we tested whether an inhibitor of viral lytic DNA replication prevented the JQ1-induced loss of RAD21 or LANA binding to KSHV genome (S2 Fig). Phosphono-acetic acid (PAA) is a potent inhibitor of KSHV lytic replication [48, 49]. While PAA inhibited KSHV genome replication by ~5 fold (S2A Fig), it did not reverse the effects of JQ1 binding on RAD21 (S2B Fig) and further stimulated the loss of LANA binding to TR (S2C Fig). We also show that induction of viral DNA replication by NaB treatment for 72 hrs, led to the loss of BRD2 or BRD4 ChIP with KSHV genome (S3 Fig). However, at 1 hr post-treatment with NaB, BRD4, but not BRD2, showed an increase binding to the lytic and latency control regions (S3C Fig). Taken together, these findings suggest that JQ1 disruption of BRD2 and BRD4 leads to a change in RAD21 and LANA binding to KSHV genome that can not be attributed to an indirect consequence of viral DNA replication.

**Fig 8:**
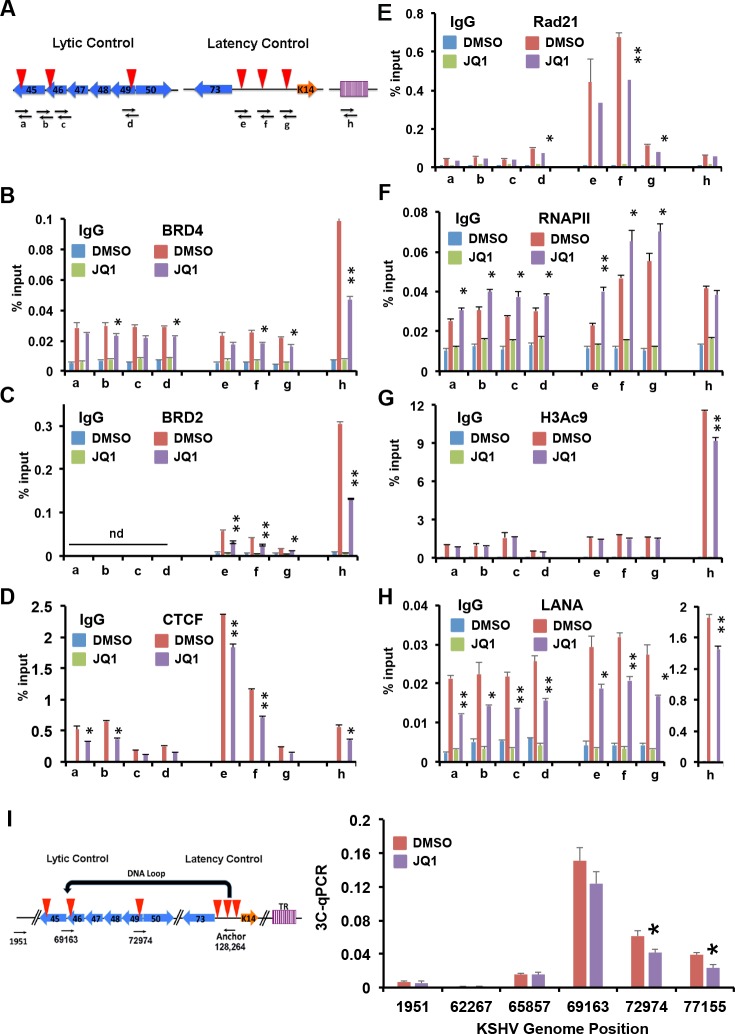
JQ1 alters epigenomic programming of KSHV latency control region. **(A)** Schematic of KSHV genome and primer positions a-h used for ChIP assays. **(B-H)** BCBL1 cells treated with 1% DMSO or 4uM JQ1 for 1 hr were assayed by ChIP for IgG or **(B**) BRD4, **(C)** BRD2, **(D)** CTCF **(E)** Rad21 **(F)** RNAPII, (**G**) H3AcK9, or (**H**) LANA. **(I)** 3C assay in BCBL1 cells treated with 1% DMSO or 4uM JQ1 for 1 hr using anchor and acceptor primers, as indicated, * P < 0.05 ** P < 0.01, nd is not determined.

To help resolve the question of whether JQ1 has a direct effect on KSHV epigenetic regulation, we assayed its effects at 1 hr post-treatment. Although this time point is too early to detect changes in KSHV transcription and DNA replication, its effect on BRD2 and BRD4 ChIP could be detected at the TR, and latency control region (Fig 8B and 8C). We also found a reduction in CTCF, RAD21, and LANA ChIP at the latency control region (Fig 8D, 8E and 8G), and a more modest loss of LANA and H3K9ac at TR (Fig 8E and 8G). We also observed a small increase in RNAPII occupancy at the latent and lytic control regions (Fig 8F). This suggests that JQ1 treatment leads to a rapid (1 hr) change chromatin regulatory factors interactions, including the loss of RAD21 and an increase of RNAPII at lytic promoters. This occurs as early as BRD2 and BRD4 disruption can be detected. To investigate whether chromosome conformation was also affected at this early time point, we performed 3C at 1 hr post-treatment with JQ1 (Fig 8I). We observed a small, but statistically significant decrease in 3C linkages at 72974 and 77153, indicating that the interaction between the latent and lytic control regions show signs of disruption at the earliest time points measured, and preceding viral lytic DNA replication.

## Discussion

**Fig 9:**
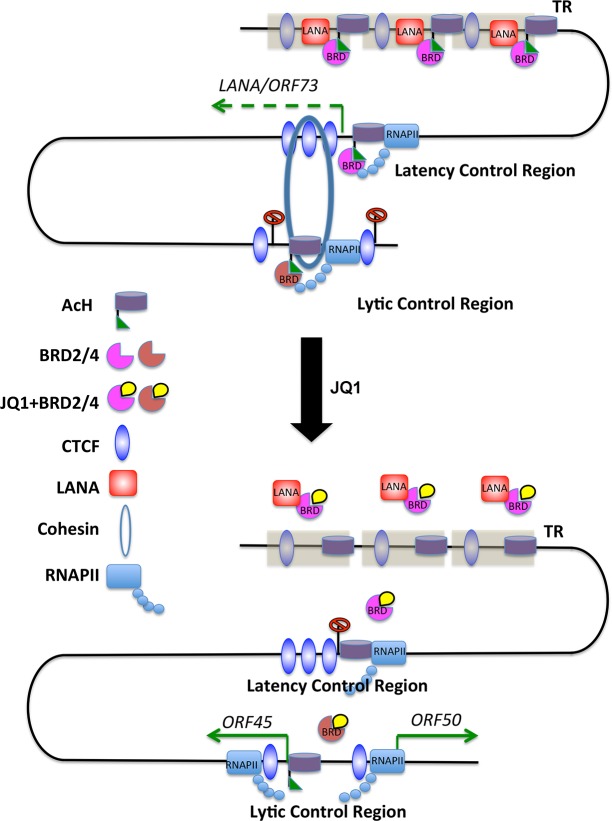
Model of JQ1 interruption of KSHV latency epigenome. Latent KSHV episomes bind BRD2 and BRD4 at sites that stabilize RNA polymerase pausing and elongation at latency control region for efficient *LANA* transcription. BRD2 and BRD4 binding to LANA protein promotes LANA binding to TR and stabilizes interactions between latent and lytic control regions. JQ1 reduces LANA binding to TR and latency control region, prevents efficient RNA pol II transcription of LANA, and destabilization DNA loop between latency and lytic control region.

Pharmacogenomics is a valuable tool for understanding biological process and pathways affected by small molecules and candidate pharmacological agents. Here, we have screened a focus library of small molecules with known inhibitory activities directed towards cellular epigenetic regulators and assayed these for their ability to stimulate KSHV lytic cycle gene expression in latently infected B-lymphoma cells. We found that bromodomain inhibitors, including JQ1, were among the more potent activators of KSHV lytic cycle gene expression. JQ1 was found to induce KSHV lytic cycle transcription, as well as DNA replication, in several different PEL cell lines. We investigated the mechanism of action of JQ1, focusing on the well-characterized JQ1 target proteins BRD2 and BRD4. BRD2 and BRD4 were found to interact with KSHV episomes at latency control regions, including the LANA binding sites in TR, and CTCF-cohesin sites at the latency and lytic control regions. Depletion of BRD2 or BRD4 partially phenocopied JQ1 activation of KSHV lytic transcription. JQ1 reduced binding of LANA to the TR and latency control region, but did not destabilize the interaction of BRD4 or BRD2 with LANA protein. JQ1 reduced RAD21 binding and disrupted a DNA loop interaction between the latent and lytic control regions. Taken together, these findings suggest that BRD2 and BRD4 contribute to maintaining the KSHV latent state, including a RAD21-dependent chromosome conformation important for KSHV latency control (Fig 9).

In contrast to our findings, others have found that JQ1 and another BET inhibitor I-BET151 show no evidence of KSHV reactivation [47, 50]. One possible explanation for these different observations is the different conditions used for JQ1 treatment. In our study, we applied 4 μM JQ1 every day for 3 days, while the previous study applied 0.5 μM once and assayed 72 hrs later. We confirmed that JQ1 can inhibit cMyc transcription as early as 6 hrs after addition of JQ1 (Figs 3 and S1). JQ1 has been shown to inhibit B-cell lymphoma proliferation by disrupting the super-enhancer activation of the cMyc gene [41, 51]. For KSHV infected PEL cells, JQ1 was found to enhance cell killing in the presence of lenalidomide which was found to selectively degrade and inhibit the IKZF1-IRF4 pathway [50]. It is possible that lenalidomide in combination with BET inhibitors prevents KSHV lytic reactivation. It is also possible that JQ1 inhibition of cMyc and other targets may trigger disruption of KSHV latency through additional indirect mechanisms. This would be consistent with the relatively slow kinetics of viral reactivation after JQ1 treatment. However, our finding that JQ1 rapidly reduces BRD2, BRD4, LANA and RAD21 interaction with KSHV genomes, and alters KSHV chromatin conformation independently of viral DNA replication, suggest that JQ1 can also act directly on the KSHV epigenome.

BET inhibitors, including JQ1, are known to activate transcription of latent forms of HIV [42]. For this reason, BET inhibitors have been considered for lytic therapy to cure latent HIV [52]. The mechanism for BET inhibitor activation of HIV may involve complicated and indirect mechanisms for BRD4 and BRD2 [42, 53]. Inhibition of BRD4 has been shown to release its interaction with 7SK repressor complex to activate RNA polymerase elongation factor pTEFb (cyclin T1 and CDK9) to drive RNA polymerase past positioned nucleosome and TAR RNA barriers [54]. In addition, BRD2 can associate with acetylated TAT protein, as well as interact with transcriptional activators and repressors that regulate HIV reactivation [53]. There are interesting parallels between HIV and KSHV latency control. Similar to HIV, KSHV latency transcription is regulated by a strongly positioned nucleosome and RNA polymerase pausing [55, 56]. The positioned nucleosome and RNA polymerase pausing depends on the cluster of CTCF binding sites in the first intron of the LANA transcript [55]. Moreover, RNA polymerase associated negative elongation factor (NELF) has been implicated in the control of KSHV lytic transcripts [57]. We found that JQ1 increased RNA polymerase II occupancy at the LANA transcript at very early times (1 hr) after treatment (Fig 8E), but this decreased at later times (72 hrs) (Fig 6D). JQ1 decreases 3C loop formation partially at early times (Fig 8I), and more significant at later times (Fig 7B). JQ1 disruption of BRD2 and BRD4 interaction with chromatin is known to occur at very early times, but how these early events regulate subsequent transcriptional and conformational events are not known. We suggest that JQ1 direct disruption of BRD2 and BRD4 interaction with KSHV chromatin leads to several subsequent events, including the loss of LANA binding to TR, loss of RAD21-dependent conformational control, and transcriptional derepression of KSHV lytic immediate early genes.

LANA is known to bind to BRD2 [58] and BRD4 [31, 32], and biophysical analyses revealed a direct interaction with the LANA DNA binding domain [30]. Interaction with BRD2/4 has been implicated in LANA metaphase chromosome tethering, as well as with transcriptional regulation [31, 32]. Our results provide evidence that inhibition of bromodomain function by JQ1 reduces LANA interaction with viral genomic DNA, suggesting that these interactions are mediated, in part, through BRD2 and BRD4 association with acetylated lysines. While LANA may have acetylated lysines [59], it is unlikely that BRD4 associated with LANA through acetyl-lysine binding, as this interaction is known to occur through the BET domain independently of the bromodomains [31]. Consistent with this, we show that JQ1 did not disrupt the interaction between LANA and BRD2 or BRD4 (Fig 5C). In contrast, JQ1 interfered with LANA binding at multiple regions of the genome, including sites that lack known consensus DNA recognition sites, such as at the latency and lytic control regions. We also observed that lytic induction by NaB reduced BRD2 binding throughout the KSHV genome (S3 Fig), but increased BRD4 binding to the latent and lytic control regions at early times after treatment (S3 Fig). This suggests that BRD2 and BRD4 have separate functions in KSHV reactivation, although additional experiments will be necessary to sort these out more precisely. BRD2 and BRD4 association with LANA is likely to facilitate LANA interaction with acetylated histones at these sites on the viral genome. This suggests that BRD2 and BRD4 facilitate LANA binding to TR in the context of chromatin, as well as target LANA to some epigenetic modifications associated with latent and lytic control regions on the viral genome.

Our findings also suggest that JQ1 disrupts the 3D conformation of the KSHV genome during latency. This disruption was reflected in the loss of 3C DNA interactions between latent and lytic control regions, as well as the reduction in Rad21 binding at the latency control region. Previous studies have indicated that Rad21 is essential for KSHV DNA conformation and loop interactions, consistent with the known function of cohesin in mediating DNA-DNA interactions [19]. LANA is also found to interact weakly with the latency and lytic control regions by ChIP assay (Fig 6F) [29, 59]. Therefore, it is possible that LANA in association with BRD2 and BRD4 mediates additional contacts between the TR and other regions of the viral genome during latency. Since JQ1 has a major effect on LANA-BRD4/BRD2 binding to TR, we propose that LANA binding at TR is important for maintaining the overall conformation of KSHV during latency (Fig 9). However, we were unable to demonstrate any direct physical interaction between BRD2 or BRD4 with cohesin subunit RAD21 (S4 Fig), suggesting the conformational control of KSHV latency involves additional factors.

High-throughput screening identified several other epigenetic modulators that may regulate KSHV lytic reactivation. We found that other BET inhibitors, including IBET-151 (Figs 3 and S1), PFI-1 and Bromosporine (general inhibitors of BRD2 and BRD4), showed low micromolar activity for KSHV reactivation. We also observed activity with several other epigenetic modulators, including inhibitors of G9A and GLP histone H3K9 methylation (UNC0638, UNC0642, and A-366). This may suggest that H3K9 methylation is an important regulator of KSHV latency in B-lymphocytes (e.g. BJAB cells) and PEL cells. This is consistent with previous studies that found peaks and valleys of H3K9me3 on the KSHV genome in latently infected BCBL1 cells [60], and may warrant future investigation. Inhibitors of EZH2 H3K27me3 methyltransferase, such as DZNep, has previously been shown to reactivate KSHV [7]. DZNep was not part of our compound library screen, and although we confirmed that H3K27me3 was enriched at lytic promoter regions (S5 Fig), we did not see any effect on H3K27me3 after BRD2 or BRD4 depletion. This suggests that BRD2 and BRD4 may function independently of the H3K27me3 associated Polycomb repression of KSHV.

The findings from this study suggest that multiple epigenetic pathways regulate KSHV latent to lytic switch. Various epigenetic modifications and processes are required to maintain the stable latent cycle gene expression program and the associated chromosome conformation. This information may provide some clinical insights into the treatment of KSHV associated disease. As JQ1 can provide a robust lytic initiating signal for latent KSHV, it may serve as an adjuvant for immune-based therapies and in combination with lytic cycle inhibitors, like gancyclovir, for pharmacological treatment of KS.

## Methods

### Cells culture and transfection

BJAB (uninfected B cell lymphoma) cells (ATCC), SLK (uninfected) cells (NIH AIDS reagent program), BJAB-BAC16 cells, KSHV positive PEL cells (BCBL1, BC3) (gift of Yan Yuan, UPENN), and double positive KSHV and EBV infected PEL cells (JSC-1, BC1) (gift of Yan Yuan, UPENN) were grown in RPMI medium (Gibco BRL) containing 10% heat-inactivated fetal bovine serum and the antibiotics penicillin and streptomycin (50U/ml). 293T cells (ATCC), iSLK (gift of J. Jung, USC and D. Ganem, Novartis) and SLK-BAC16 were cultured in Dulbecco’s modified Eagle’s medium with 10% fetal bovine serum and the antibiotics. iSLK cells were cultured in the presence of 1 μg/ml puromycin and 250 μg/ml G418. KSHV BAC16 and its derivatives were introduced into iSLK cells via Fugene HD transfection as described previously [61]. Two days post transfection, iSLK-BAC16 cell lines were established and maintained in the presence of 1ug/ml puromycin, 250ug/ml G418, and 1,000ug/ml hygromycin B. For transient transfection, actively growing 293T cells were processed with Lipofectamine reagent (Invitrogen), and the cells were harvested 72 hours post transfection. All cells were cultured at 37°C in a 5% CO_2_ environment.

### KSHV BAC16 virus production and infection

Stable iSLK-BAC16 cells were induced in the presence of both doxycycline (1 μg/ml) and sodium butyrate (1 mM) and the absence of hygromycin, puromycin, and G418. Four days later, supernatant was collected and cleared of cells and debris by centrifugation (1,000 g for 15 min at 4°C) and filtration (0.45 μm). Virus particles were pelleted in 25% sucrose/1x PBS solution by ultracentrifugation (100,000 g for 1 h at 4°C). BJAB and SLK cells were infected with concentrated KSHV-BAC16 viruses derived from induced iSLK-BAC16 cells as described above. BJAB cells were subjected to spin infection for 30 min at 450 x g and 25°C in the presence of 8 ug/ml of polybrene (Sigma). SLK cells were seeded at approximately 2x10^5^ cells/well in 6 well plate 24h prior to infection and then inoculate overnight. After 48 hrs, cells were selected by hygromycin at 200ug/ml for 2 weeks and KSHV episome presence was checked by PFGE. Derived stable lines were designated as BJAB-BAC16 or SLK-BAC16 cells.

### Plasmids

KSHV LANA was cloned into p3XFLAG-CMV-24 (Sigma) as described previously [59]. Human BRD4 and BRD2 expression constructs were a gift from Dr. Jianxin You (University of Pennsylvania, School of Medicine).

### Generation of shRNA lentiviruses and lentivirus infection

Lentiviruses were generated and lentivirus infection were performed as described previously [16]. PEL cells were harvested at 6 or 10 days post puromycin selection. shRNA for BRD2 (TRCN0000006308 and 6309) and BRD4 (TRCN0000021427 and 21428) were obtained from Sigma TRC library.

### Compound screening of epigenetic library

A library of 24 compounds with known epigenetic targets were arrayed in 10-point dose response analysis from 20μM to 1nM. Row B17-B22 and O3-O8 received 2 μM sodium butyrate and 50 μg/ml TPA as positive control for lytic reactivation, and the remaining wells of row B and O received DMSO (0.2% final concentration).

BJAB-BAC16 cells were generated by infecting BJAB cells with recombinant BAC16 KSHV derived virus and maintained in RPMI with 10% FBS. 50 μl containing 25,000 BJAB-BAC16 cells were dispensed using a Biotek Microflo into clear 384-well tissue culture plates (Greiner Inc., cat# 781–192). Fifty nanoliters per well of test compound was transferred to assay plates using a Janus MDT equipped with a 384 nanohead (Perkin-Elmer Inc). Cells were incubated with compounds for 48 h at 37°C in 5% CO_2_ incubator. To determine the lytic population, 10,000 GFP-positive live cells were analyzed by fluorescence activated flow cytometry (FACS) using a high-throughput sampler attached to a BD FACSCalibur (BD Biosciences). The gate for the lytic population was set by the increment of GFP intensity during lytic reactivation. The percentage of lytic cells was acquired from this gate for data analysis. Similarly, cells were analyzed to by an Operetta high content scanner (Perkin Elmer, Inc) with a 20x objective. For Operetta analysis, assay plates were prepared by incubating 5,000 BJAB-BAC16 cells with compounds in 50 μl of RPMI at 37°C in 5% CO_2_. Plates were centrifuged at 250g for one minute and 3 fields per well were subsequently imaged at 24h and 48h post drug treatment. The acquired GFP fluorescent images were analyzed using Harmony software and GFP intensity per cell was determined. Data of test compounds was normalized to DMSO controls to calculate fold lytic activation (i.e., fold lytic activation = test (compound)/average(DMSO)). EC50 values were determined using Spotfire (TIBCO, Perkin-Elmer) data analysis. Active compounds of interest for further study were defined as those with a reproducible EC50 of less than 1μM in activating lytic replication. A Z’-factor was calculated to measure the statistical relevance of the screen, where Z’ equals (1 − 3(σ_p_ − σ_n_)/|μ_p_ − μ_n_|) where σ is variance, μ is mean, with p representing positive controls, and n negative.

### ChIP

Chromatin immunoprecipitation (ChIP) assays were performed as described previously [19]. Antibodies used in the ChIP assays are listed below. Primers for ChIP assays were used as described previously [19]. PCR data were normalized to input values that were quantified in parallel for each experiment.

### Antibodies and reagents

The following antibodies were used for ChIP assays: anti-IgG (Santa Cruz Biotechnology), anti-CTCF (Millipore), anti-Rad21 (Abcam), anti-acetylated H3K9 (Millipore), RNA polymerase II (Santa Cruz sc-889x), anti-BRD2 (Bethyl), anti-BRD4 (Bethyl) antibodies. The mouse monoclonal antibody anti-IgG (Santa Cruz Biotechnology) and Rat anti-KSHV LANA antibody (Advanced Biotechnologies Inc.) were used for ChIP assays. Rabbit polyclonal anti-BRD4 (Bethyl), mouse monoclonal anti-actin (Sigma) and anti-FLAG (Sigma) antibodies were used for Western blotting. JQ1 was a gift from the Jay Bradner Lab, I-BET151 from Sigma-Aldrich and BIC1 from Calbiochem and were used at a concentration of 4 uM. Phosphonoacetic acid (PAA) was purchased from Sigma and used at a concentration of 400 ug/ml.

### Quantification of viral intracellular DNA.

The amount of intracellular KSHV DNA was determined by quantitative PCR (qPCR) analysis of purified total genomic DNA as described previously [19].

### IP

Immunoprecipitation (IP) was performed as described previously [62].

### RT-qPCR

RT-PCR was performed as described previously [19].

### PFGE and southern analysis

BCBL1 cells infected with lentivirus expressing shControl, shBRD2 or shBRD4 were used for PFGE and PFGE was performed as described previously [19]. Hirt DNA extraction and Southern analysis were performed as described previously [19]. KSHV DNA was quantified by PhorphorImager.

### Statistical analysis

p-values were calculated by 2-tailed student t-test using Excel (Microsoft, Redmond, WA). * p<0.05, ** p<0.01, *** p<0.001.

## Supporting Information

S1 Fig BET inhibitors induce KSHV lytic reactivation in BC3 cells. RT-qPCR for c-myc, LANA, ORF50 and PAN relative to actin in BC3 cells treated with 1%DMSO, 4uM JQ1, or 4uM IBET-151 for the indicated times. The data are expressed as fold change of the JQ1 or I-BET151 versus untreated (DMSO) cells. * P < 0.05 ** P < 0.01 *** P < 0.001.
(TIF)

S2 Fig Effects of viral lytic replication on JQ1 inhibition of RAD21 and LANA binding. **(A)** qPCR of KSHV genome in BCBL1 cells treated daily with DMSO, JQ1 (4 μM), or JQ1 (4 μM) + PAA (400 μg/ml) for 72 hrs. The data are expressed as fold change of the JQ1 + PAA treated versus JQ1 treated cells. *** P < 0.001. **(B)** ChIP assay for RAD21 in BCBL1 cells treated as described in panel A. **(C)** ChIP assay for LANA in BCLB1 cells treated as described in panel A. ChIP DNA was analyzed at KSHV genome with primers sets described in Fig 5A. * P < 0.05 ** P < 0.01.
(TIF)

S3 Fig Sodium Butyrate (NaB) alters BRD2 and BRD4 binding to KSHV genome. **(A)** Schematic of KSHV genome and position of primers used for ChIP assay. (**B -D)** BCBL1 cells were treated with DMSO control, JQ1 (4 μM) or NaB (2 mM) for 1 hr (panels **B** and **C**) or 72 hrs (panels **D** and **E**) and then assayed by ChIP with antibody to either BRD2 (panels **B** and **D**) or BRD4 (panel **C** and **E**). * P < 0.05 ** P < 0.01.
(TIF)

S4 Fig Failure to CoIP BRD2 or BRD4 with RAD21. BCBL1 cells were treated with DMSO or JQ1 for 1 hr and then processed for IP with antibody to RAD21 or IgG and then assayed by Western blot with antibody to RAD21, BRD4, BRD2, or SMC1 (left panel). Similarly, BCBL1 cells were processed for IP with either BRD2, BRD4, or IgG and assayed by Western blot with antibody for RAD21, BRD4, BRD2, or LANA (right panel). While RAD21 could coIP with SMC1, it did not coIP with BRD2 or BRD4. Similarly, while BRD4 could coIP with LANA it did not coIP with RAD21 or BRD2. BRD2 did not coIP with LANA, BRD4, or RAD21.
(TIF)

S5 Fig H3K27me3 is elevated at KSHV lytic control region but not affected by shRNA depletion of BRD2 or BRD4. **(A)** KSHV genome and primer positions for ChIP assay. **(B)** BCBL1 cells transduced with shControl, shBRD2, or shBRD4 were subject to ChIP assay with antibody to IgG or H3K27me3. While H3K27me3 is elevated at lytic control region (primers a-d), the depletion of BRD2 or BRD4 did not affect H3K27me3 levels. shBRD2 and shBRD4 depletion was from material shown in Fig 4.
(TIF)
