# Supplementary figures and images for: BET-Inhibitors Disrupt Rad21-Dependent Conformational Control of KSHV Latency
Source: PLoS Pathog. 2017 Jan 20;13(1):e1006100. doi: 10.1371/journal.ppat.1006100 (PMC5287475; doi:10.1371/journal.ppat.1006100)

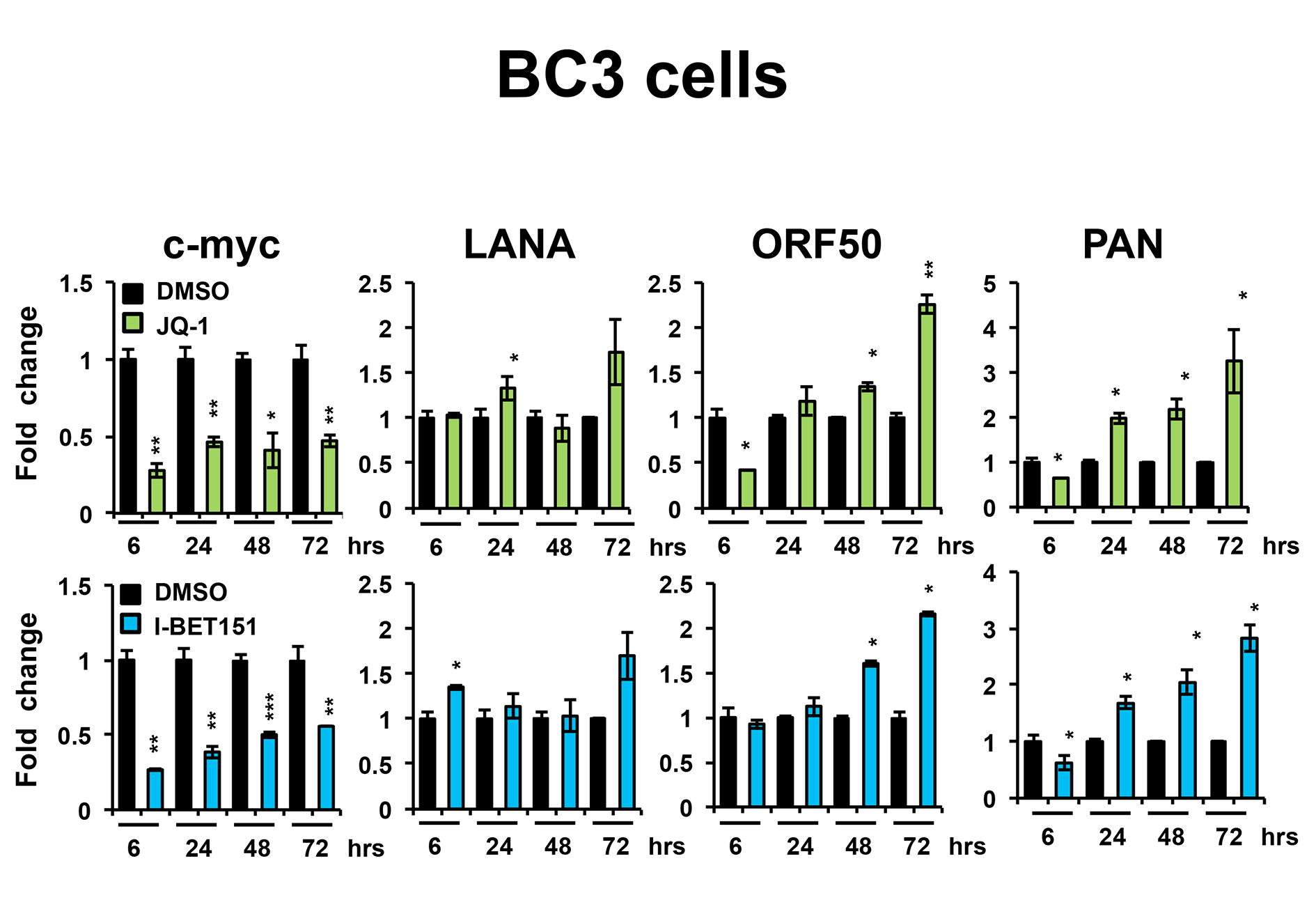

Supplement: S1 Fig — RT-qPCR for c-myc, LANA, ORF50 and PAN relative to actin in BC3 cells treated with 1%DMSO, 4uM JQ1, or 4uM IBET-151 for the indicated times. The data are expressed as fold change of the JQ1 or I-BET151 versus untreated (DMSO) cells. * P < 0.05 ** P < 0.01 *** P < 0.001. (TIF) [file ppat.1006100.s001.tif]

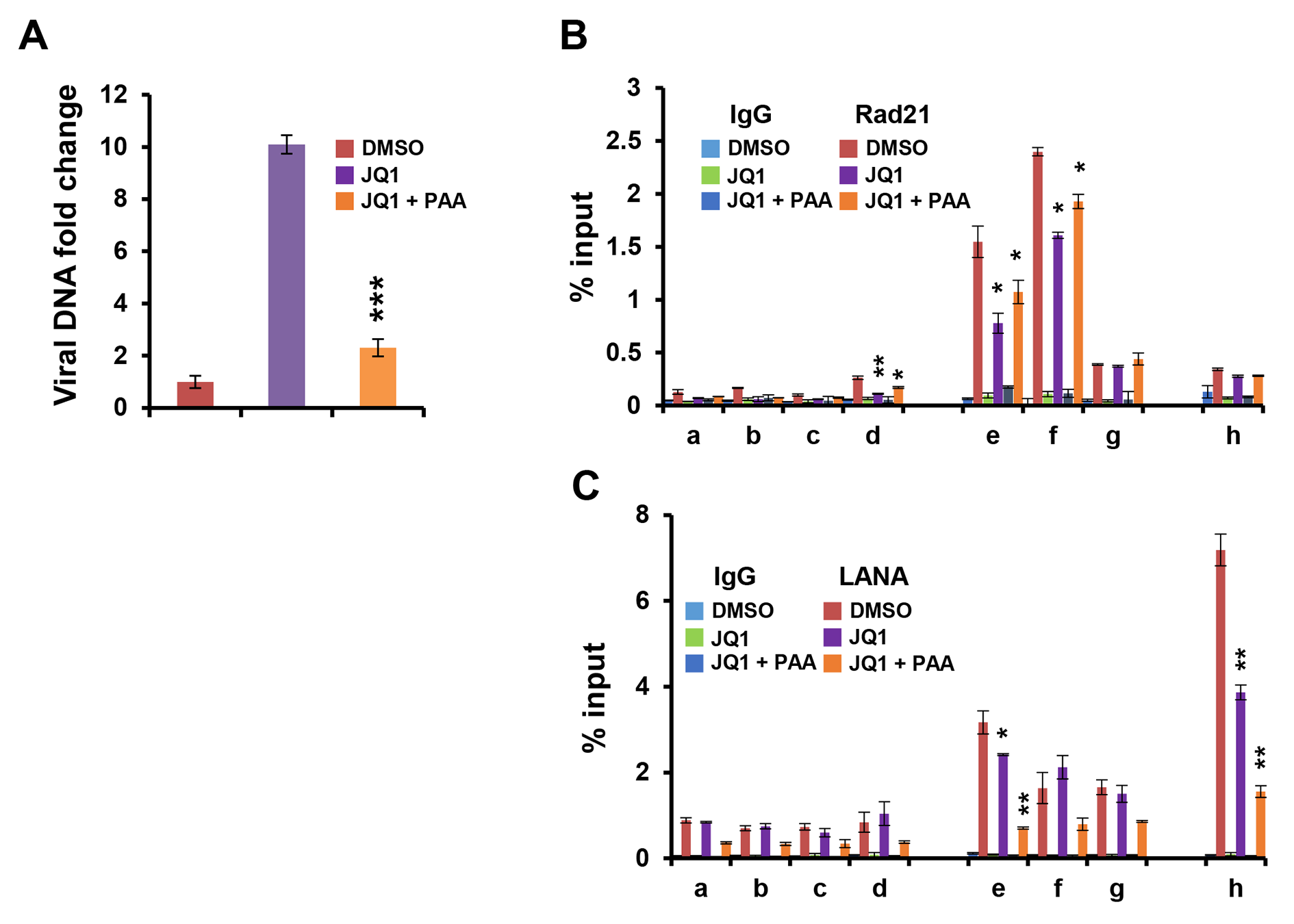

Supplement: S2 Fig — (A) qPCR of KSHV genome in BCBL1 cells treated daily with DMSO, JQ1 (4 μM), or JQ1 (4 μM) + PAA (400 μg/ml) for 72 hrs. The data are expressed as fold change of the JQ1 + PAA treated versus JQ1 treated cells. *** P < 0.001. (B) ChIP assay for RAD21 in BCBL1 cells treated as described in panel A. (C) ChIP assay for LANA in BCLB1 cells treated as described in panel A. ChIP DNA was analyzed at KSHV genome with primers sets described in Fig 5A. * P < 0.05 ** P < 0.01. (TIF) [file ppat.1006100.s002.tif]

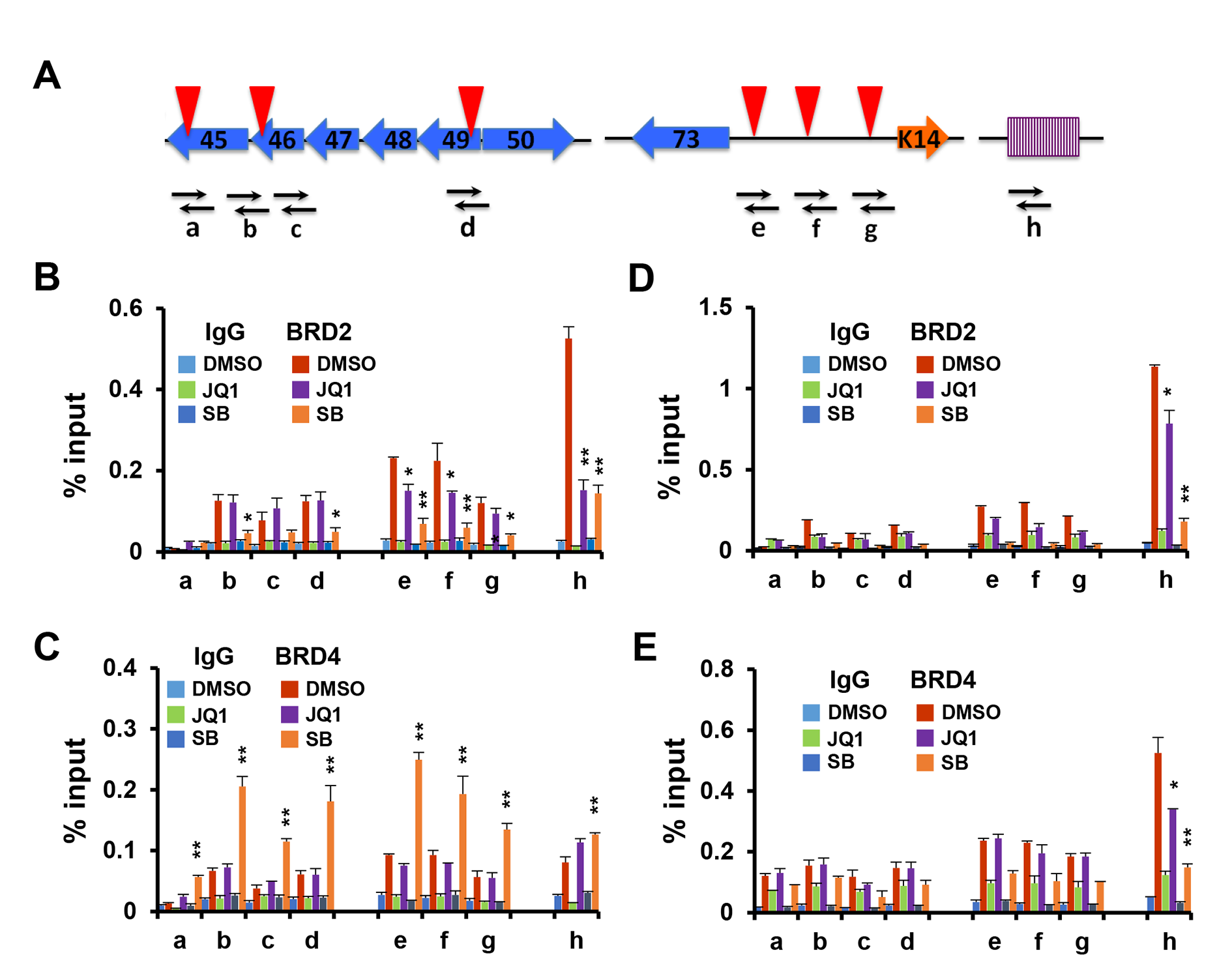

Supplement: S3 Fig — (A) Schematic of KSHV genome and position of primers used for ChIP assay. (B -D) BCBL1 cells were treated with DMSO control, JQ1 (4 μM) or NaB (2 mM) for 1 hr (panels B and C) or 72 hrs (panels D and E) and then assayed by ChIP with antibody to either BRD2 (panels B and D) or BRD4 (panel C and E). * P < 0.05 ** P < 0.01. (TIF) [file ppat.1006100.s003.tif]

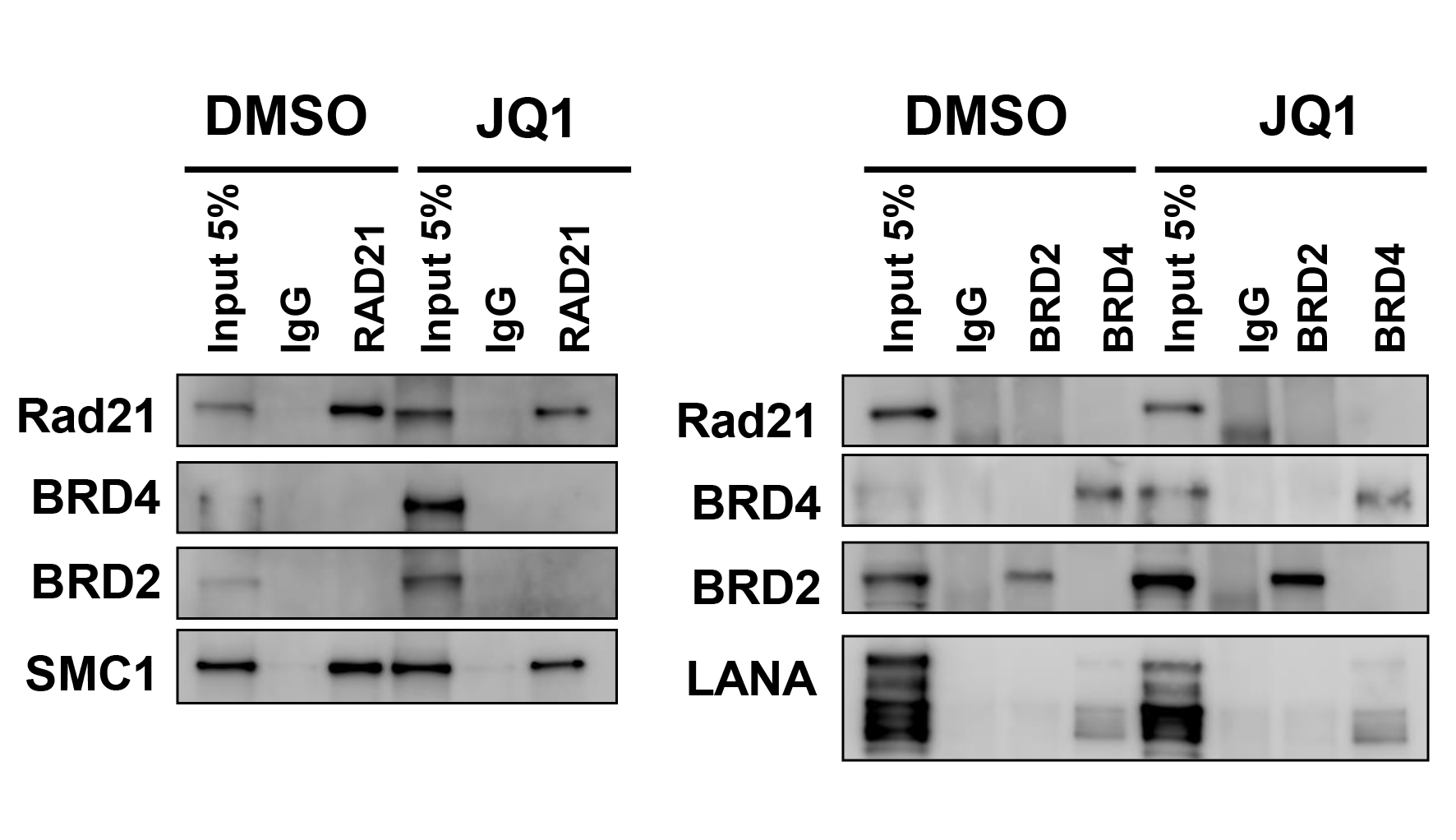

Supplement: S4 Fig — BCBL1 cells were treated with DMSO or JQ1 for 1 hr and then processed for IP with antibody to RAD21 or IgG and then assayed by Western blot with antibody to RAD21, BRD4, BRD2, or SMC1 (left panel). Similarly, BCBL1 cells were processed for IP with either BRD2, BRD4, or IgG and assayed by Western blot with antibody for RAD21, BRD4, BRD2, or LANA (right panel). While RAD21 could coIP with SMC1, it did not coIP with BRD2 or BRD4. Similarly, while BRD4 could coIP with LANA it did not coIP with RAD21 or BRD2. BRD2 did not coIP with LANA, BRD4, or RAD21. (TIF) [file ppat.1006100.s004.tif]

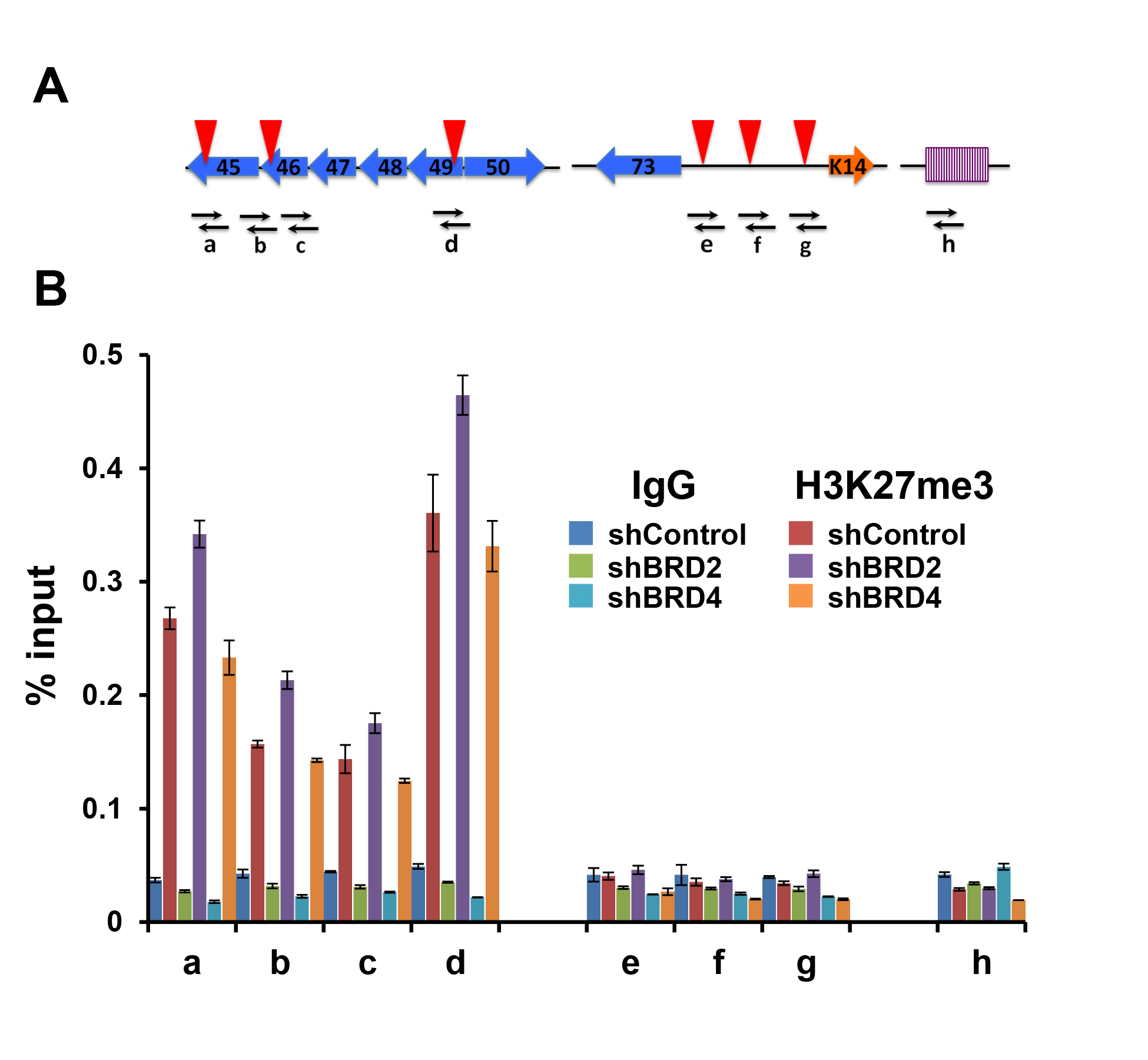

Supplement: S5 Fig — (A) KSHV genome and primer positions for ChIP assay. (B) BCBL1 cells transduced with shControl, shBRD2, or shBRD4 were subject to ChIP assay with antibody to IgG or H3K27me3. While H3K27me3 is elevated at lytic control region (primers a-d), the depletion of BRD2 or BRD4 did not affect H3K27me3 levels. shBRD2 and shBRD4 depletion was from material shown in Fig 4. (TIF) [file ppat.1006100.s005.tif]
